# Comparing Hydraulics Between Two Grapevine Cultivars Reveals Differences in Stomatal Regulation Under Water Stress and Exogenous ABA Applications

**DOI:** 10.3389/fpls.2020.00705

**Published:** 2020-06-19

**Authors:** Silvina Dayer, Johannes D. Scharwies, Sunita A. Ramesh, Wendy Sullivan, Franziska C. Doerflinger, Vinay Pagay, Stephen D. Tyerman

**Affiliations:** ^1^School of Agriculture, Food and Wine, The University of Adelaide, Glen Osmond, SA, Australia; ^2^Australian Research Council Centre of Excellence in Plant Energy Biology, Waite Research Institute, School of Agriculture, Food and Wine, The University of Adelaide, Adelaide, SA, Australia

**Keywords:** *Vitis vinifera*, gas exchange, aquaporin, plasma membrane intrinsic protein, tonoplast intrinsic protein, hydraulic conductivity, isohydric, isohydrodynamic

## Abstract

Hydraulics of plants that have different strategies of stomatal regulation under water stress are relatively poorly understood. We explore how root and shoot hydraulics, stomatal conductance (*g*_s_), leaf and root aquaporin (AQP) expression, and abscisic acid (ABA) concentration in leaf xylem sap ([ABA]_xylemsap_) may be coordinated under mild water stress and exogenous ABA applications in two *Vitis vinifera* L. cultivars traditionally classified as near-isohydric (Grenache) and near-anisohydric (Syrah). Under water stress, Grenache exhibited stronger adjustments of plant and root hydraulic conductances and greater stomatal sensitivity to [ABA]_xylemsap_ than Syrah resulting in greater conservation of soil moisture but not necessarily more isohydric behavior. Correlations between leaf (Ψ_leaf_) and predawn (Ψ_PD_) water potentials between cultivars suggested a “hydrodynamic” behavior rather than a particular iso-anisohydric classification. A significant decrease of Ψ_leaf_ in well-watered ABA-fed vines supported a role of ABA in the soil-leaf hydraulic pathway to regulate *g*_s_. Correlations between leaf and root AQPs expression levels under water deficit could explain the response of leaf (*K*_leaf_) and root (*Lp*_r_) hydraulic conductances in both cultivars. Additional studies under a wider range of soil water deficits are required to explore the possible differential regulation of *g*_s_ and plant hydraulics in different cultivars and experimental conditions.

## Introduction

To withstand abiotic stresses such as drought, plants have evolved complex adaptive mechanisms that are regulated dynamically. There is an interplay between stomatal regulation of transpiration (Chaves et al., 2010) and changes in the hydraulic conductivity of roots [*Lp*_r_; (Maurel et al., 2010)], and leaves [*K*_leaf_; Sack and Holbrook (2006)]. The transpiration rate of leaves, or water flux through stomata, is regulated by stomatal conductance (*g*_s_) and strongly influenced by vapor pressure deficit [VPD; Aphalo and Jarvis (1991); McAdam et al. (2016)] as well as changes in *K*_leaf_ (Pou et al., 2013), through changes of stomatal guard cell turgor. Changes in guard cell turgor involve complex and still debated mechanisms that are mediated by chemical and/or hydraulic signals (Comstock, 2002). It is well established that under soil and/or atmospheric water deficit (WD), i.e., soil drying or high VPD, respectively, abscisic acid (ABA), a plant growth regulator, is synthesized in the leaves of plants (McAdam et al., 2016), where it induces stomatal closure (Schroeder et al., 2001; Dodd, 2003, 2005). Initially it was thought that ABA acts as a long distance signal from roots to the shoot during abiotic stress. However, Christmann et al. (2007) demonstrated that signals other than ABA might be involved in this process and supporting the role of hydraulics in modulating stomatal responses to WD. Recently, a root peptide signal was discovered in Arabidopsis during soil moisture deficit, that could induce ABA synthesis in the leaves (Takahashi et al., 2018).

Even though ABA signaling is seen as the main pathway for stomatal regulation, chemical signals other than ABA have been proposed to contribute significantly (Christmann et al., 2007; Wilkinson et al., 2007) including the recently reported γ-aminobutyric acid [GABA; Mekonnen et al. (2016)]. Christmann et al. (2007) demonstrated in *Arabidopsis thaliana* that changes in turgor pressure of leaf mesophyll cells occurred within minutes of root-induced osmotic stress and elicited activation of ABA biosynthesis and signaling required for stomatal closure. These observations support a role of ABA in stomatal closure but call into question whether it acts as the sole primary-long distance signal of water stress.

Hydraulic mediation of stomatal closure has been observed in studies where large diurnal fluctuations of *g*_s_ and leaf water potential (Ψ_leaf_) were observed without substantial changes in the soil water content (Salleo et al., 2000). The co-variation of *g*_s_ and Ψ_leaf_ has been interpreted as a mechanism to protect the plant from severe dehydration and consequently, xylem cavitation and loss of hydraulic conductivity (Tyree and Sperry, 1989). Other studies have suggested the presence of hydraulic signals based on positive correlations between *K*_lea__f_ and *g*_s_ at a relatively constant Ψ_leaf_ (Nardini et al., 2001). Evidence for the involvement of a hydraulic root-to-shoot signal has been provided by experiments where wild-type tomato plants were grafted on ABA-deficient roots (Holbrook et al., 2002). Despite the inability of the mutant roots to produce increased amounts of ABA during WD, the stomata still showed the wild-type response to WD. Recent studies in grapevine suggested that *g*_s_ was regulated to a greater degree by hydraulic rather than chemical signals during the early phases of WD, while ABA seemed to have an additive effect involved in the long-term maintenance of stomatal closure under prolonged WD (Tombesi et al., 2015). According to these studies, the involvement of both hydraulic and chemical signals seems to be a more likely explanation in the regulation of *g*_s_ under WD.

Aquaporins (AQPs), cellular membrane-bound water channel proteins and members of the major intrinsic protein (MIP) family, have been shown to play a key role in the transcellular or radial flow of water in both leaves and roots (Steudle, 2000; Chaumont and Tyerman, 2014). AQPs are known to be regulated by cytoplasmic pH, divalent cations, and phosphorylation (Tournaire-Roux et al., 2003; Tornroth-Horsefield et al., 2006; Li et al., 2015). In certain species, the transcellular path is a major contributor to *K*_leaf_ (Prado and Maurel, 2013). Rapid and reversible changes in *K*_leaf_ involving AQPs have been observed under fluctuating environmental conditions such as solar radiation (Prado et al., 2013), WD (Galmes et al., 2007), and in response to exogenous application of ABA (Shatil-Cohen et al., 2011; Pantin et al., 2013). For example, in grapevine, *K*_leaf_ decreased by about 30% under water stress concomitantly with a decrease of expression of some plasma membrane intrinsic protein (PIP) and tonoplast intrinsic protein (TIP) AQP isoforms (Pou et al., 2013). Furthermore, the same study found significant positive correlations between *g*_s_, *K*_leaf_ and leaf AQP expression suggesting a contribution of AQPs in regulating the flow of water. In *Arabidopsis*, xylem-fed ABA reduced *K*_leaf_ by specifically decreasing the water permeability of vascular bundle sheath cells, putatively through inactivation of PIPs (Shatil-Cohen et al., 2011). In line with that study, Pantin et al. (2013) confirmed those observations and proposed a model in which ABA closes stomata via its already known chemical effect on guard cells (Tardieu and Simonneau, 1998) as well as via an indirect hydraulic action by decreasing leaf water permeability within vascular tissues (Pantin et al., 2013). According to these findings, the hydraulic signal induced by ABA may be an important component in the mechanisms used by different species to regulate *g*_s_ under WD.

In addition to *g*_s_ and *K*_leaf_ variations, responses to drought include changes in root hydraulic conductivity (*Lp*_r_). In contrast to the commonly observed reduction in *K*_leaf_, ABA application and WD usually have opposite effects on *Lp*_r_: while water stress reduces *Lp*_r_, most studies have reported increased *Lp*_r_ with ABA (Aroca et al., 2006; Thompson et al., 2007; Parent et al., 2009). The increase in *Lp*_r_ by ABA can be interpreted as a mechanism to improve the water supply to the shoot, helping to maintain the water continuum in the plant under soil or atmospheric WD (Kudoyarova et al., 2011; Pantin et al., 2013). Diurnal changes in *Lp*_r_ have been observed under well-watered (WW) conditions concomitantly with changes in shoot transpiration (Vandeleur et al., 2009). In general, these variations correlate with the transcript abundance of root AQPs suggesting that water transport across roots is regulated by AQPs to meet the transpirational demand of the shoots (Sakurai-Ishikawa et al., 2011; Laur and Hacke, 2013; Vandeleur et al., 2014). Accordingly, these studies support the hypothesis of shoot-to-root (chemical and/or hydraulic) signaling via the xylem that regulates *Lp*_r_ in response to *E* that is modulated by AQPs (Vandeleur et al., 2014). Positive correlations between *Lp*_r_, *g*_s_, and *E* have also been observed in grapevines exposed to exogenous ABA applications through the soil suggesting a connection between ABA-mediated root and leaf conductances (DeGaris, 2016).

The hydraulic and chemical (ABA-mediated) mechanisms described above that operate between roots and leaves to control *g*_s_ may be important in understanding the contrasting behaviors described previously in the literature, namely “isohydry” or “anisohydry” for various species and even cultivars (varieties or sub-species) to cope with WD (Pantin et al., 2013). Near-isohydric plants have been reported to maintain Ψ_leaf_ relatively constant under declining soil moisture availability through a tight regulation of *g*_s_ (Tardieu and Simonneau, 1998). This behavior has been reported to confer an advantage of increased drought tolerance (Schultz, 2003) and is thought to be under hydraulic and chemical (ABA) control (Tardieu and Simonneau, 1998). In contrast, it has been suggested that near-anisohydric plants maintain *g*_s_ to prioritize photosynthesis, which is related to a more variable Ψ_leaf_ (Tardieu and Simonneau, 1998). This behavior has been previously reported to operate under chemical control (Tardieu et al., 1996).

The present study aimed to elucidate how the hydraulic behavior, gas exchange and aquaporin expression in two grapevine cultivars previously classified as near-isohydric (Grenache) and near-anisohydric (Syrah) (Schultz, 2003) differed in their responses to a mild WD, recovery from WD, and exogenous ABA application to roots. We hypothesized that under mild WD or exogenous ABA application, the more “isohydric” Grenache would decrease *Lp*_r_ concomitantly with *g*_s_ to maintain homeostasis of Ψ_leaf_ mediated by a down-regulation of leaf and root AQPs. In contrast, the relatively anisohydric Syrah would maintain *Lp*_r_ under mild WD through an up-regulation of root AQPs in order to maintain homeostasis of *g*_s_, but Ψ_leaf_ is expected to decrease.

## Materials and Methods

### Experimental Site and Plant Material

The experiments were carried out in 2015 and 2016 at The Plant Accelerator^®^, The University of Adelaide, Waite Campus located in Urrbrae (Adelaide), South Australia (34° 58′ 17′′ S, 138° 38′ 23′′ E). One-year-old rootlings of own rooted grapevines (*Vitis vinifera* L.) cvs. Grenache and Syrah were planted in 4.5 L pots containing a mixture of 50% vermiculite and perlite and 50% of UC soil mix (61.5 L sand, 38.5 L peat moss, 50 g calcium hydroxide, 90 g calcium carbonate) and 100 g Nitrophoska^®^ (12:5:14, N:P:K plus trace elements; Incitec Pivot Fertilisers, Southbank, Vic., Australia) per 100 L at pH 6.8. Plants were grown for 2 months in a temperature-controlled glasshouse (day/night: approx. 25/20°C) and irrigated to field capacity every 3 days from December 21st 2015. The vines were pruned to two shoots 10 days after bud burst (January 10th, 2016) and oriented upright during their development using wooden stakes. A liquid soil fertilizer, Megamix (13:10:15 N:P:K plus trace elements; Rutec, Tamworth, NSW, Australia), was applied at a concentration of 1.6 mL L^–1^ to bring all plants to approximately equal size. The fertilizer was applied weekly for 3 weeks once the plants had developed the first adult leaves. On the 25th February 2016, all vines were moved from the greenhouse and transferred to a DroughtSpotter (Phenospex, Netherlands) automated gravimetric watering platform where individual pots were automatically weighed continuously (15 min intervals) and watered twice daily (0600 h, 1600 h) based on the weight loss by transpiration. All plants were irrigated to their field capacity mass (determined the previous days) daily until the start of the experiment. Day (16 h) and night (8 h) temperatures in the DroughtSpotter glasshouse were kept at 25/20°C, respectively.

### Treatments

Grenache and Syrah vines were used to examine the effects of WD and recovery by re-watering (REC) on *g*_s_ and root hydraulic conductivity (*Lp*_r_; normalized to root dry weight). A set of vines were kept as control (well-watered; WW), irrigated to field capacity by weight to replace the amount of water consumed by transpiration daily. WD was imposed starting on Day 4 by reducing the amount of irrigation until a moderate WD (target *g*_s_ ∼ 50 mmol H_2_O m^–2^ s^–1^) was reached on Day 5 (Medrano et al., 2002). After 3 days of WD at approx. the target *g*_s_, on Day 8, the vines were rehydrated by irrigating the pots to field capacity and recovery from water stress was examined after 7 days (Day 14). An additional treatment consisting of an exogenous application of ABA was simultaneously carried out on a separate set of vines from both cultivars. In this treatment, the vines were root-fed by applying 50 μM of ABA (Valent Biosciences Corporation, Libertyville, IL, United States) daily to the root system concurrently with irrigation. This concentration of ABA was based on prior experiments, which showed that 50 μM of ABA applied to the root system of potted vines is required to have a significant effect on *g*_s_ (DeGaris, 2016). All the pots were covered with a thick layer of perlite to minimize soil water loss through evaporation.

On the night before each measurement day, due to space constraints in the DroughtSpotter glasshouse, randomly selected vines from each treatment were moved from the DroughtSpotter glasshouse to an adjacent larger glasshouse that was at the same environmental conditions for physiological measurements and tissue sampling for gene expression analysis. The three measurement days were: (i) Day 5 for the WW and WD vines, when the WD vines reached a target *g*_s_ of between 50 and 100 mmol H_2_O m^–2^ s^–1^; (ii) Day 7 for the WW, WD, and ABA-treated vines; and, (iii) Day 14 on the WW and REC vines, 7 days after rewatering commenced in the REC vines. At each time point, three to five WW vines were used as controls to compare against the specific treatment(s).

### Physiological Measurements

#### Leaf Gas Exchange

In order to track *g*_s_, daily measurements of *g*_s_ were performed in all vines on the DroughtSpotter platform on fully expanded leaves (estimated minimum leaf age: Leaf Plastochron Index, LPI >10) in the basal section of the shoots. These daily measurements were performed mid-morning (1030–1130 h) daily on two fully expanded and mature leaves per vine with a porometer (SC-1, Decagon Devices, Pullman, WA, United States). In an earlier characterization of the performance of the porometer, we observed a strong correlation of *g*_s_ measured by the porometer and *g*_s_ measured using an open system infrared gas analyzer (LI-COR, Model 6400XT).

On the specific sampling dates, *g*_s_ and *E* were measured concomitantly with other physiological measurements between 1000 h and 1100 h. Measurements were performed on two fully expanded, healthy leaves using an open system infrared gas analyzer (IRGA; LI-6400XT, LI-COR Biosciences Inc., Lincoln, NE, United States) with a 6 cm^2^ cuvette. An external LED light source (LI-6400-02B) attached to the cuvette was used at a fixed PAR value of 1500 μmol m^–2^ s^–1^ due to the non-saturating light levels in the glasshouse for photosynthesis (approx. 200 μmol m^–2^ s^–1^). After gas exchange measurements were performed, the same leaf was excised to determine Ψ_leaf_.

#### Leaf Water Potential and Sap Collection for ABA Analysis

Predawn, leaf and stem water potentials were measured on mature, primary leaves of vines on the specific sampling dates. Predawn leaf water potential (Ψ_PD_) was measured before sunrise (0400–0500 h), and leaf (Ψ_leaf_) and stem (Ψ_stem_) water potentials around midday (1100–1200 h). Ψ_stem_ was measured after leaves equilibrated in aluminum foil and plastic bags for 2 h. One leaf per vine was measured from three to five vines per group using a Scholander-type pressure chamber (PMS Instruments Co., Albany, OR, United States).

After recording leaf water potential values, an overpressure of ∼0.5 MPa was applied to the encapsulated leaf for xylem sap collection (approx. 35 μL). The sap was collected from the cut surface of the protruding petiole using a micropipette and transferred to a pre-weighed and labeled micro tube before snap freezing in liquid nitrogen. Samples were stored at −80°C until subsequent analysis of ABA.

#### ABA Analysis of Xylem Sap

Abscisic acid concentration in xylem sap samples ([ABA]_xylemsap_) was analyzed as described in Speirs et al. (2013). Briefly, the volume of each sample was measured using a pipette for normalization. Each sample was mixed with 30 μL of deuterated standard (Plant Biotechnology Institute, Saskatoon, SK, Canada) containing deuterium-labeled analogs of ABA, phaseic acid (PA), dihydrophaseic acid (DPA) and the glucose ester of ABA (ABA-GE) at a concentration of 100 ng mL^–1^ each. Solids were precipitated in a centrifuge at 12,470 × *g* for 5 min. From each sample, 20 μL supernatant was transferred to a LC/MS tube and analyzed by liquid chromatography/mass spectrometry (Agilent 6410 Triplequadropole LC-MS/MS with Agilent 1200 series HPLC, Agilent Technologies Inc., Santa Clara, CA, United States). A Phenomenex C18(2) column (75 mm × 4.5 mm × 5 μm; Phenomenex, Torrance, CA, United States) was used at 40°C and samples were eluted with a 15 min linear gradient of 10–90% acetonitrile. Nanopure water and acetonitrile were both mixed with 0.05% acetic acid. Compounds were identified by retention times and mass/charge ratio.

#### Hydraulic Conductance of Leaves, Plant and Roots

Leaf and whole plant hydraulic conductances (*K*_leaf_ and *K*_plant_) were determined using the evaporative flux method (Sack et al., 2002; Flexas et al., 2013). This measurement is based on the relationship between the leaf transpiration rate (*E*) and the water potential gradient (ΔΨ) when leaf water potential (Ψ_leaf_) reaches a steady state. In this case hydraulic conductance is calculated as follows: *K*_l__eaf_ = *E*/(Ψ_stem_–Ψ_leaf_) and *K*_plant_ = *E*/(Ψ_PD_–Ψ_leaf_). Our approach of estimating hydraulic conductances using measurements of *E* with an IRGA (leaf enclosed in a cuvette) rather than with a flowmeter or gravimetrically (on an unenclosed leaf) relies on the assumption that the leaf boundary layer resistance is negligible, which is addressed by adequate mixing of the air inside the cuvette.

The hydraulic conductance of the entire root system (*Lp*_*r*_) was measured for the same plants using a High Pressure Flow Meter (HPFM; Dynamax, Houston, TX, United States) as previously described in Vandeleur et al. (2009). This is a destructive technique whereby the stem of the vine is cut above the soil surface, covered with filtered deionized water and the stump connected to the HPFM with a water-tight seal as quickly as possible, typically within 1 min. A transient ramp in pressure (up to 0.5 MPa at a rate of 7 kPa s^–1^) with simultaneous recording of flow rate was used to calculate hydraulic conductance, which was normalized by dividing by total root dry weight to obtain *Lp*_r_. Since the hydraulic conductance is inversely proportional to the viscosity of water, the measured conductance was temperature-corrected internally within the HPFM. All measurements were conducted within 5 min of shoot excision. The soil was washed from the roots before drying at 60°C for more than 48 h prior to weighing.

#### AQPs Expression in Roots and Leaves

Samples of leaves and roots were collected from vines immediately after physiological measurements for subsequent analysis of AQP transcript abundance by quantitative reverse transcription PCR (RT-qPCR). Leaves were immediately immersed in liquid nitrogen and stored at −80°C until analysis. Roots were carefully selected from the bottom and upper parts of the pot in order to get the thinner, white and more functional roots. The root samples were quickly washed to remove soil particles and dried with tissue paper before being submerged in liquid nitrogen.

Leaf and root material was ground to a fine powder in liquid nitrogen using a mortar and pestle. For leaves, total RNA was extracted from 100 mg of fine frozen powder using the Spectrum Plant Total RNA extraction Kit (Sigma-Aldrich, St. Louis, MO, United States). DNA contamination was avoided by digestion with the On-Column DNase I Digestion Set (Sigma-Aldrich, St. Louis, MO, United States) during RNA extraction according to manufacturer recommendations. For roots, RNA extractions were performed as described by Vandeleur et al. (2014). RNA was extracted from 200 mg of fine frozen powder with a 20 mL sodium perchlorate extraction buffer (5 M sodium perchlorate, 0.2 M Tris pH 8.3, 8.5% (w/v) polyvinylpolypyrrolidone, 2% PEG 6000, 1% (w/v) SDS, 1% (v/v) β-mercapto-ethanol) for 30 min at room temperature. The lysate was filtered through a glass wool filter and mixed with 30 mL of cold absolute ethanol before precipitation at −20°C overnight. After centrifugation at 3500 rpm for 20 min at 4°C, the pellets were washed with cold ethanol and purified using the Spectrum Plant Total RNA Extraction Kit with on-column DNase digestion as described for leaves. Concentration and purity of total RNA were determined on a NanoDrop^TM^ 1000 Spectrophotometer (Thermo Fisher Scientific Inc., Waltham, MA, United States). Agarose gel electrophoresis (1.2% agarose) was done to visualize the integrity of RNA.

For cDNA synthesis, 1 μg of total RNA was reverse transcribed using iScript^TM^ cDNA Synthesis Kit for RT-qPCR (Bio-Rad, Hercules, CA, United States) as per the manufacturer’s instructions.

Gene expression analysis was carried out by quantitative real time PCR (Bio-Rad iCycler iQ system; Bio-Rad) in a 20 μL mixture containing 1 μL of 1:10 diluted cDNA, 10 μL iQ SYBR Green Reaction-Mix (Bio-Rad), 0.6 μL of each primer, and 2.7 μL of DEPC water. Three biological and technical replicates were run for each sample. Thermal cycling conditions were as follows: one cycle of 30 s at 95°C followed by 40 cycles of 20 s at 95°C, 20 s at 59°C, and 20 s at 72°C. A previous standard quantification curve with five serial dilutions of cDNA was constructed for each gene to calculate amplification efficiency. The fluorescence threshold value (Ct) was calculated using the iCycle iQ system software (Bio-Rad). Overall, a mean Ct value was calculated from three independent biological replicates, each with three PCR replicates. Elongation factor (*ELF*), *GAPDH*, actin and ubiquitin were examined as possible reference genes across the treatments using the software tool NormFinder (Molecular Diagnostic Laboratory, Department of Molecular Medicine, Aarhus University Hospital, Skejby, Denmark; Andersen et al., 2004; Selim et al., 2012). Of these, ELF was the most stable and hence used for normalization. Gene expression was relative to mean of the control calculated as (*E*_target_) DCT_target_ (control–sample)/ (*E*_ref_) DCT_ref_ (control–sample) (Pfaffl, 2001), where *E*_t__arget_ and *E*_ref_ are the efficiencies of the target gene and reference gene (ELF), respectively, determined from a dilution series. The absence of non-specific products was confirmed by both the analysis of the melt curves and by electrophoresis in 2% (w/v) agarose gel of the PCR product. The primer sequences used for the amplifications were designed by Vandeleur et al. (2009) based on published sequences of AQPs found in grapevine (Supplementary Table S1).

### Statistical Analyses

All statistical analyses and preparation of figures were performed in the statistical language R (R Core Team). To compare differences between treatments (WW, WD, ABA, REC) and cultivars (Grenache, Syrah), linear models of the form *Y ∼ Treatment × Cultivar* were fitted to the data and analysis of variance (ANOVA) was performed to test for general differences among groups. For pairwise comparisons, estimated marginal means were obtained using the R package “emmeans” (Lenth, 2019) and *p*-values were adjusted based on the multivariate *t* distribution (adjust = “mvt”). Significant differences (*P* ≤ 0.05) are indicated by different letters in the figures and described in the text. Both linear and non-linear models were fitted to the data for correlations between two continuous variables. Comparisons of two linear regression models were performed by analysis of covariance (ANCOVA).

## Results

### Water Relations of a Mild Water Deficit and ABA Watered Vines

**FIGURE 1:**
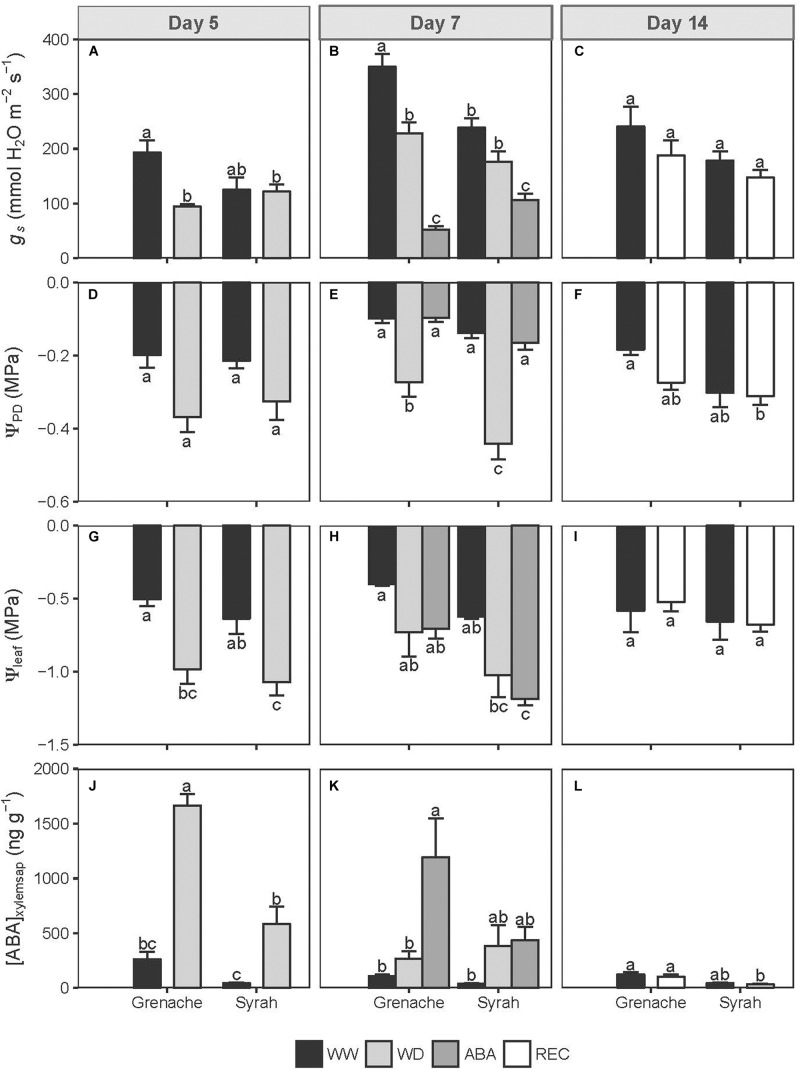
Stomatal conductance (*g*_s_; **A–C**), pre-dawn (Ψ_PD_; **D–F**) and leaf (Ψ_leaf_; **G–I**) water potentials and ABA concentration in the xylem sap ([ABA]_xylemsap_; **J–L**) in Grenache and Syrah grapevines under mild water deficit (WD), exogenous application of abscisic acid (ABA) and recovery from water stress (REC) at different sampling days along the experiment. Values are means ± SE (*n* = 5). Different letters indicate statistically significant differences (*P* ≤ 0.05) across all treatments and cultivars within the day.

A soil moisture deficit imposed on the two cultivars in this study, Syrah and Grenache, resulted in similar values of Ψ_PD_ on Day 5 for the WD vines (Figure 1D) and a range of Ψ_PD_ values from −0.2 to −0.5 MPa were measured in WD vines for Days 5 and 7 (Figures 1D,E). However, by Day 7, 3 days after the onset of stress, a significantly lower Ψ_PD_ was measured for WD vines of Syrah compared to Grenache (Figure 1E). This deficit resulted in the WD vines of Grenache showing a reduction in *g*_s_ on Day 5 while Syrah did not show this trend (Figure 1A). On Day 7, WD vines from both cultivars showed a similar reduction of *g*_s_ relative to WW plants after the deficit was sustained for 3 days (Figure 1B), however, this difference was only significant for Grenache. WD vines of both Grenache and Syrah showed a similar reduction in leaf water potential (Ψ_leaf_) on Days 5 and 7 (Figures 1G,H). In comparing the two cultivars, the WD vines of Syrah had, on average, a lower Ψ_leaf_ compared to Grenache (Figure 1H). Syrah showed a trend toward higher vine water stress based on its lower Ψ_leaf_.

The ABA concentrations in the xylem sap were significantly higher in the WD vines from both cultivars relative to the controls, but Grenache presented a higher concentration than Syrah on Day 5 (Figure 1J). On Day 7, the average ABA concentration in the xylem sap was higher in WD and ABA treated vines compared to WW vines, but only ABA treated Grenache had a significantly higher [ABA]_xylemsap_ concentration (Figure 1K). ABA-treated vines showed a significant reduction of *g*_s_ as compared to the controls as well as the WD vines (Figure 1B) despite adequate soil moisture and similar values of Ψ_PD_ than WW vines (Figure 1E). For either cultivar, ABA-treated vines had a similar Ψ_leaf_ to that of WD vines (Figure 1H). When WD vines were recovered (REC) for 7 days, they showed no significant differences in vine water status or [ABA]_xylemsap_ compared to the WW vines (Figures 1C,F,I,L).

### Correlations Between Physiological Variables

**FIGURE 2:**
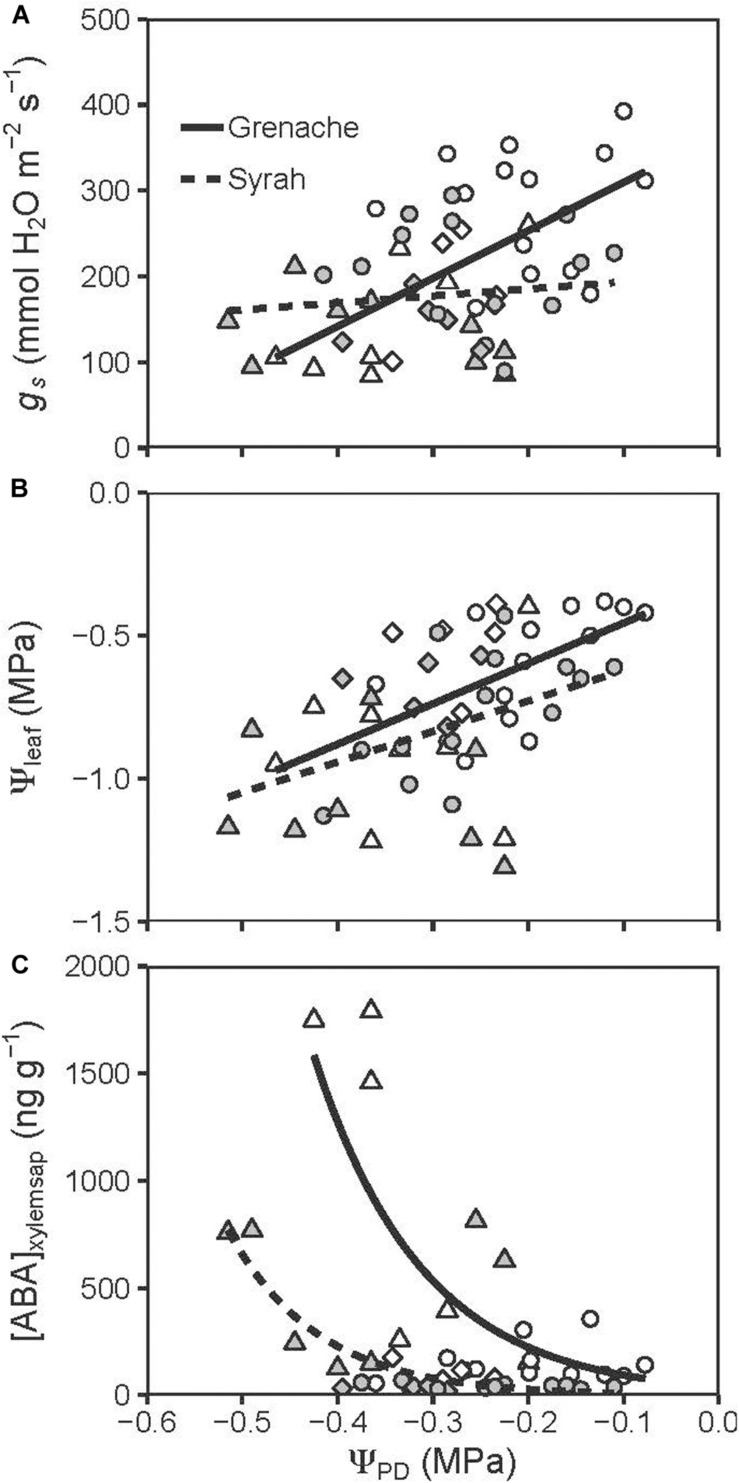
Correlation between Ψ_PD_ and *g*s **(A)**, Ψ_leaf_ **(B)**, and [ABA]_xylemsap_ **(C)** for well-watered (circles), water deficit (triangles), and recovery from water stress vines (diamonds) from the cultivars Grenache (open symbols) and Syrah (filled symbols). **(A)** Grenache and Syrah show a significantly different (*P* = 0.017) relationship between Ψ_PD_ and *g*_s_: in Grenache *g*_s_ is significantly positively correlated to Ψ_PD_ (*r* = 0.58, *P* = 0.002) while no correlation was found for Syrah (*r* = 0.14, *P* = 0.487). **(B)** For both cultivars Ψ_leaf_ is significantly positively correlated to Ψ_PD_ [Grenache: *r* = 0.54, *P* = 0.004; Syrah: *r* = 0.44, *P* = 0.021]. No significant differences were found for the slope (*P* = 0.580) and intercept (*P* = 0.091) of the linear regression lines [Grenache: Ψ_leaf_ (MPa) = 1.4 ± 0.4 × Ψ_PD_ (MPa) –0.3 ± 0.1; Syrah: Ψ_l__eaf_ (MPa) = 1.1 ± 0.4 × Ψ_PD_ (MPa) –0.5 ± 0.1]. **(C)** Non-linear regressions between Ψ_PD_ and [ABA]_xylemsap_ for Grenache ([ABA]_xylemsap_ = 39.1 ± 60.4 ng g^–1^ × exp[–8.7 × ± 4.3 (1/MPa) × Ψ_PD_ (MPa)]) and Syrah ([ABA]_xylemsap_ = 3.3 ± 5.7 ng g^–1^ × exp[–10.5 × ± 3.5 (1/MPa) × Ψ_PD_ (MPa)]).

A closer examination between some physiological variables showed a significantly (*P* = 0.017) stronger response of *g*_s_ to changes in Ψ_PD_ in vines of Grenache compared to vines of Syrah, when Ψ_PD_ dropped from approx. −0.1 MPa to −0.5 MPa (Figure 2A). In contrast, Ψ_leaf_ declined similarly in response to decreasing Ψ_PD_ in both cultivars (Figure 2B). Different responses of stomata between cultivars were in line with differences observed for the relationship between [ABA]_xylemsap_ and Ψ_PD_ (Figure 2C). Grenache had a steeper response to a decrease in the Ψ_PD_ than Syrah with a significant increase in [ABA]_xylem_ values below Ψ_PD_ of −0.3 MPa. In contrast, Syrah had a lower and later increment of [ABA]_xylemsap_ showing values lower than 1000 ng g^–1^ over the range of Ψ_PD_ values measured in this study. The ABA catabolites, PA and DPA, were found to increase in the xylem sap of ABA-treated and WD vines compared to WW vines, which was apparent as early as Day 5 (Supplementary Figure S3). These catabolites decreased in concentration upon recovery from water stress on Day 14 to levels below those of WW vines.

**FIGURE 3:**
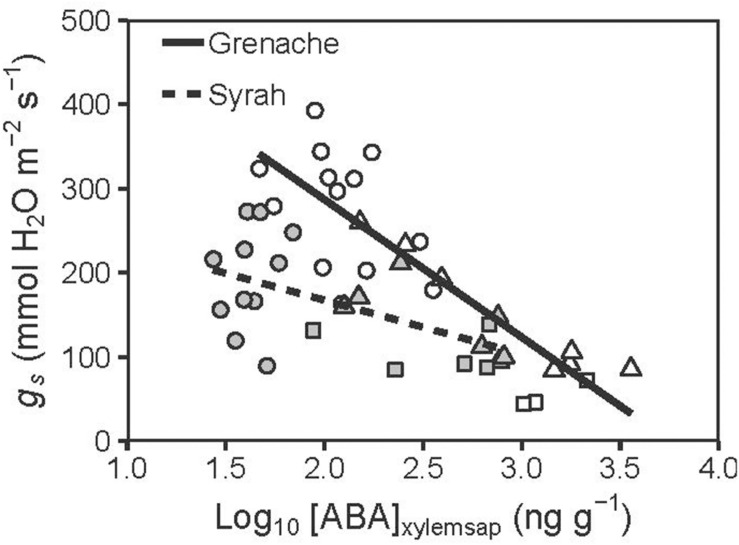
Relationship between [ABA]_xylemsap_ (Log_10_ transformed) and *g*_s_ measured for well-watered (circles), water deficit (triangles), and ABA treated vines (squares) from the cultivars Grenache (open symbols) and Syrah (filled symbols). Significant negative regressions were predicted [Grenache: *g*_s_ = –163.4 ± 20.9 × log_10_([ABA]_xylem_) + 613.6 ± 53.1; *r* = –0.86] and Syrah [*g*_s_ = –63.9 ± 19.6 × log_10_([ABA]_xylem_) + 295.2 ± 42.7; *r* = –0.58]. Both linear regressions had significantly different slopes (*P* = 0.001) and significantly different intercepts (*P* < 0.001).

The two cultivars were compared for the relationship between *g*_s_ and [ABA]_xylemsap_ in Figure 3. The decline in *g*_s_ with increasing [ABA]_xylemsap_ for Syrah (slope: −63.9 ± 19.6 mmol H_2_O m^–2^ s^–1^ ng^–1^ g) was significantly (*P* = 0.001) lower than that for Grenache (slope: −163.4 ± 20.9 mmol H_2_O m^–2^ s^–1^ ng^–1^ g) indicating a higher stomatal sensitivity of Grenache to ABA compared to Syrah. Interestingly, vines that were treated with exogenous ABA (instead of WD) showed the same relationship between [ABA]_xylemsap_ of leaves and *g*_s_ for both cultivars; these data are included as square symbols in Figure 3.

**FIGURE 4:**
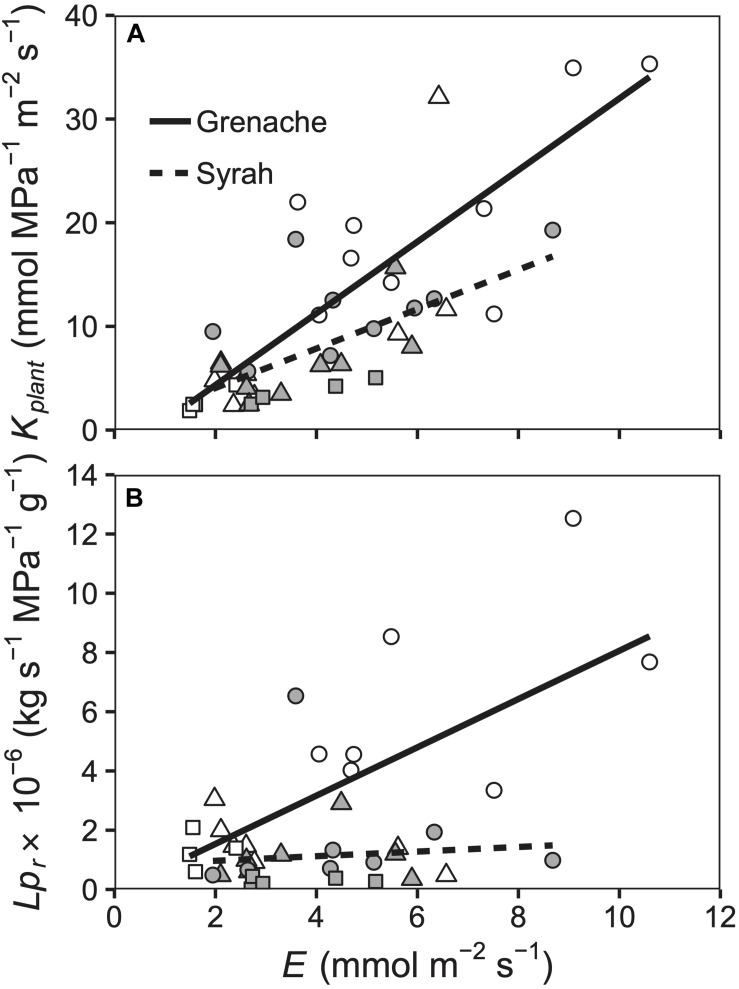
Relationship between *E* and *K*_plant_ **(A)** and *Lp*_r_ **(B)** for well-watered (circles), water deficit (triangles), and ABA treated vines (squares) for the cultivars Grenache (open symbols) and Syrah (filled symbols). **(A)** *K*_plant_ was positively correlated to *E* for both Grenache [*K*_plant_ = 3.5 ± 0.5 × E – 2.6 ± 2.7; *r* = 0.84] and Syrah [*K*_plant_ = 1.9 ± 0.5 × E + 0.3 ± 2.4; *r* = 0.63]. The slopes were not significantly different. **(B)** Lpr had a significant different (*P* = 0.027) relationship to *E* in the cultivar Grenache [*Lp*_r_ = 0.8 ± 0.2 × E – 0.1 ± 1.1; *r* = 0.69] compared to the cultivar Syrah [*Lp*_r_ = 0.1 ± 0.2 × E + 0.8 ± 0.9; *r* = 0.09].

In comparing leaf and root hydraulics, both *K*_plant_ and *Lp*_r_ decreased in parallel with decreased transpiration (*E*), but there were significant differences between Grenache and Syrah when considering *Lp*_r_ only (Figure 4). As compared to whole plant hydraulic conductance (*K*_plant_), *Lp*_r_ of Grenache changed significantly in response to different levels of *E* compared to vines from the cultivar Syrah (Figure 4B). It should be noted that *K*_plant_ was determined using values of *E* obtained from an IRGA and therefore expected to show a strong relationship with *E* as observed in Figure 4A.

Leaf hydraulic conductance (*K*_leaf_) was observed to decrease significantly in Grenache, but not in Syrah, in response to exogenous ABA application through the soil water (Supplementary Figure S4). Withholding water from the soil did not significantly lower *K*_leaf_ in either cultivar.

**FIGURE 5:**
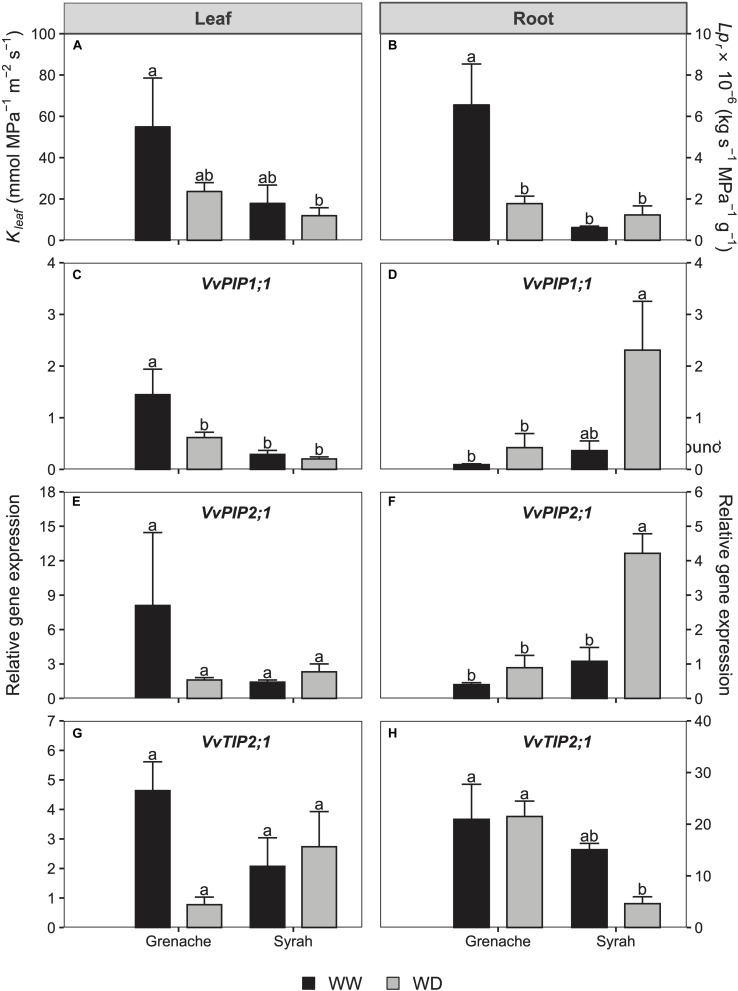
Leaf (*K*_leaf_; **A**) and root (*Lp*_r_; **B**) hydraulic conductances and gene relative expression of *PIP1*;*1*, *PIP2*;*1*, and *TIP2*;*1* in the leaf **(C,E,G)** and roots **(D,F,H)** in Grenache and Syrah grapevines under well-watered (WW) and mild water deficit (WD) at Day 5 of the experiment. Values are means ± SE (*n* = 3). Different letters indicate statistically significant differences (*P* ≤ 0.05) across both treatments and cultivars.

On Day 5, leaf hydraulic conductance (*K*_leaf_) was observed to decrease in WD Grenache vines, but not in Syrah, however, these differences were not statistically significant, likely due to the small sample number and scatter in the data (Figure 5A and Supplementary Figure S4). Similarly, root hydraulic conductance (*Lp*_r_) decreased significantly in the WD vines compared to the control vines in Grenache, but not in Syrah (Figure 5B).

### Do Expression Levels of Certain AQPs Correlate With Changes in Hydraulic Conductance?

Several AQPs in the leaves and roots of both cultivars were analyzed by qPCR. These included: *PIP1*;*1*, *PIP2*;*1*, *PIP2*;*2*, *PIP2*;*3*, *TIP1*;*1*, and *TIP2*;*1*. Of these AQPs, only *PIP1;1* (Figures 5C,D), *PIP2;1* (Figures 5E,F), and *TIP2;1* (Figures 5G,H) showed differences between the treatments so are discussed in this paper. The decrease in *K*_l__eaf_ observed on Day 5 in Grenache compared to Syrah was associated with some of the AQPs expressed in the leaves. For instance, the expression of *PIP1*;*1* in leaves was down-regulated under WD conditions in Grenache, but not in Syrah (Figure 5C). In the roots, the decrease in *Lp*_r_ observed in Grenache under WD was not related to changes in AQPs levels. In contrast, *PIP2*;*1* was up-regulated in Syrah without any changes in *Lp*_r_ (Figure 5F). AQPs were also analyzed in ABA-treated grapevines, however, no differences were found between WW and ABA vines in any of the AQPs evaluated.

## Discussion

The traditionally so-called isohydric behavior of plants has been characterized by a limited decline in Ψ_leaf_ by the closure of stomata under WD, while anisohydric behavior has been characterized by maintenance of high *g*_s_ and consequently a greater reduction in Ψ_leaf_. There is much interest in these different behaviors since agriculturally important plants with different degrees of stomatal regulation can have very different water demands under certain environmental conditions and therefore require different irrigation management strategies. However, the hydraulic and gas exchange properties of plants that confer either near-isohydry or anisohydry are still relatively poorly understood and controversies found in recent studies have argued that stomatal regulation is a response to the plant-environment interaction rather than a genetically determined trait (Hochberg et al., 2017; Charrier et al., 2018).

In this study, we investigated specific mechanisms that may explain the differences in iso/anisohydry, in particular, root hydraulics, leaf gas exchange, leaf and root AQP expression, and xylem ABA. We used two *V. vinifera* cultivars, Grenache and Syrah, which have previously been shown to exemplify the differences in isohydry (Schultz, 2003). Our choice of cultivars, Grenache and Syrah, have been used as models for near-isohydric and anisohydric classification, respectively, in several studies (Schultz, 2003; Soar et al., 2006; Prieto et al., 2010; Coupel-Ledru et al., 2014, 2017; Gerzon et al., 2015; Scharwies and Tyerman, 2016; Shelden et al., 2017).

To impose water stress on the vines, our experiment aimed for a target *g*_s_ of 50 mmol H_2_O m^–2^ s^–1^ as this represents a moderately severe degree of plant water stress based on downregulation of net photosynthesis and electron transport rate as reported in Medrano et al. (2002). We acknowledge that this approach has limitations including the possibility of different perceptions of stress (at this *g*_s_ level) by different genotypes. There are similar drawbacks using soil moisture thresholds for drought stress experiments as different genotypes would likely have different plant responses, e.g., hydraulic and chemical signaling, to any level of soil moisture.

### Stomatal Sensitivity to ABA

In the present study, water-stressed Syrah vines presented a significantly lower Ψ_PD_ and Ψ_leaf_ on Day 7 of the experiment compared to Grenache that would indicate a more anisohydric behavior. However, the response of *g*_s_ to WD was only different between cultivars on Day 5 when Grenache showed an earlier reduction of *g*_s_ to WD than Syrah, indicating a more isohydric behavior, as expected (Figure 2A). However, this level of stomatal closure in Grenache was insufficient to maintain a homeostasis of Ψ_leaf_ as might be expected with isohydric behavior. It is important to note that only a mild WD was imposed in this experiment with Ψ_PD_ values not lower than −0.6 MPa and that measurements were performed on different days and thus different environmental conditions (e.g., VPD; Supplementary Figure S1). In this regard, recent studies have suggested that the iso/anisohydric classification is a continuum behavior rather than a genetic trait possessed by a cultivar and that it depends on the level of drought imposed and prevailing VPD conditions (Charrier et al., 2018). In that study, the authors observed in four different cultivars a threshold of Ψ_PD_ (∼−0.7 MPa) at which the stomata become more sensitive to VPD, and this response was similar for all cultivars. In our study, we did not achieve that Ψ_PD_ threshold so it is possible that the *g*_s_ response to Ψ_PD_ observed here might not account for small differences in VPD between different days (Supplementary Figure S1).

Despite this, the mild WD imposed here reveals differences in some physiological variables among cultivars, but not necessarily a more isohydric behavior. For instance, a higher sensitivity of *g*_s_ to changes in Ψ_PD_ and ABA was observed in Grenache compared to Syrah (Figures 2A, 3, respectively). In addition, higher [ABA]_xylemsap_ was produced by Grenache leaves at decreasing water potentials than Syrah indicating a higher sensitivity of this cultivar to changes in soil water potential as well as a greater reliance on chemical signaling from roots to shoots (Figure 2C). These results are in agreement with other studies confirming a higher sensitivity of *K*_leaf_ to ABA fed via the petiole in Grenache compared to Syrah (Coupel-Ledru et al., 2017). However, Tardieu and Simonneau (1998) suggested that an isohydric behavior could be related to stomatal sensitivity to ABA being modulated by Ψ_leaf_ or *E*, i.e., an indirect response of *g*_s_ to ABA (e.g., via leaf AQPs), while anisohydric behavior could be due to a direct response of *g*_s_ to ABA.

Our observations of the ABA response on *g*_s_ was as expected: *g*_s_ decreased markedly on Day 7 in both cultivars in ABA-fed vines compared to WW vines (Figure 1B). Interestingly, we also measured a significant decrease of Ψ_leaf_ in Syrah, but not in Grenache, in response to ABA feeding (Figure 1H), which suggests a hydraulic involvement, i.e., water potential and/or hydraulic conductance, in Syrah. This potential role of ABA in stomatal regulation via hydraulics rather than directly as a chemical signal has been previously mentioned in Arabidopsis (Pantin et al., 2013). The down-regulation of several AQPs in response to ABA and the decrease of hydraulic conductance in the roots, as shown in Figure 4B (square symbols), could be associated with this response.

Based on the assumption that ABA acted directly or indirectly on *g*_s_, differences in the response of *g*_s_ to WD observed in the two cultivars may be due to Grenache producing more ABA or Syrah having a higher rate of ABA catabolism. The maximum ABA concentration in the xylem sap of leaves measured during the experiment in all treatments was higher in Grenache compared to Syrah (Figure 1). Rossdeutsch et al. (2016) found that the average ABA concentration in the xylem sap of Grenache shoots during drought stress was similar to Syrah shoots, however, the same authors measured significantly higher concentrations of DPA, a degradation product of ABA, in Syrah compared to Grenache. This may indicate that Syrah has a higher catabolism of ABA, which may lower its hypersensitivity to WD relative to Grenache, and results of the present study support this hypothesis. We observed that, on Day 7, water-stressed Syrah vines had nearly three-fold higher DPA concentration in the xylem sap of leaves compared to WW vines, similar to the difference of DPA between WW and WD Grenache vines on Day 5 (Supplementary Figure S3). The higher DPA levels suggest greater cumulative water stress in Syrah compared to Grenache as ABA catabolism results in accumulation of DPA and PA over time (Cutler and Krochko, 1999). ABA could also act indirectly on Ψ_leaf_ and decrease its value by decreasing *K*_leaf_, which has been proposed by Shatil-Cohen et al. (2011). This putative reduction in *K*_leaf_ occurs as water flow through the symplastic (cell-to-cell) pathway via leaf bundle sheath cells decreases. Future research could aim to test the stomatal sensitivity to ABA in Grenache and Syrah at different and lower Ψ_leaf_ values to verify the hypothesis by Tardieu and Simonneau (1998).

### Hydraulic Response to Mild WD: Any Divergent Strategy?

Regulation of hydraulic conductivities in roots, stems and leaves has been used previously to determine the iso-anisohydric cultivar behavior. Schultz (2003) suggested differences in *K*_leaf_ between Grenache and Syrah could be the origin of their isohydric and anisohydric behavior, respectively. In the present study, *K*_leaf_ was higher in Grenache which could be explained by its larger xylem vessels (Gerzon et al., 2015; Shelden et al., 2017) as compared to Syrah, however, xylem vessel sizes were not measured in this study. Different correlations between gas exchange parameters and hydraulic conductances between cultivars suggest that the hydraulic pathway in some plants could be more adaptive to changes in *E*, which is an important consideration since physiological adaptations of the hydraulic pathway are expected to occur over longer time scales than the evolution of water stress in the present study. For instance, significant differences in the response of *Lp*_r_ to changes in *E* separated the two cultivars (Figure 4B). This is an important new finding since little is known about root hydraulics in characterizing cultivars with expected contrasting behaviors (Lovisolo et al., 2010). Distinct differences in *Lp*_r_ have previously been linked to different expression patterns of AQPs hypothesized to result from a xylem-mediated hydraulic signal, possibly from shoots to roots (Vandeleur et al., 2014). The linear regressions between *E* and *Lp*_r_ calculated in this study for Grenache were similar to the linear regressions shown by Vandeleur et al. (2009). Interestingly, Coupel-Ledru et al. (2017) found that *K*_leaf_ of detached leaves fed with solutions of different ABA concentrations decreased with increasing ABA concentration in Grenache, but no change in *K*_leaf_ was observed in Syrah. This response is similar to what we observed in Grenache leaves (Supplementary Figure S4), as well as roots which showed a stronger response than Syrah to changes in *E* (Figure 4B). Evidence of decreased *K*_leaf_ in response to ABA in Grenache in the present trial supports the finding that Ψ_leaf_ was similar between the WD vines of the two cultivars (Figure 1H) despite significantly different Ψ_PD_ (Figure 1D). This suggests that ABA had the effect of increasing the leaf hydraulic resistance or decreasing *K*_leaf_. This decrease in *K*_leaf_ is not unexpected as it was determined using an IRGA via measurements of *E*. Future experiments could confirm this finding using independent measures of *K*_leaf_ for example with the evaporative flux (gravimetric or flow-based) or rehydration kinetics methods (Flexas et al., 2013).

Simonin et al. (2015) suggested that a positive correlation between *E* and *K*_leaf_ could help to stabilize the gradient between Ψ_stem_ and Ψ_leaf_ for hydraulic transport to the leaf. This would cause less variation in Ψ_leaf_, which is a feature of isohydric behavior to maximize *g*_s_ and, therefore, CO_2_ uptake for photosynthesis. At the whole plant level, our results showed no significant changes in the gradient between Ψ_PD_ and Ψ_leaf_ (i.e., ΔΨ_plant_) in relation to changes in *E* for both cultivars despite differences in hydraulic regulation (Supplementary Figure S2), a behavior termed “isohydrodynamic” by Franks et al. (2007). Furthermore, considering the correlation between Ψ_PD_ and Ψ_leaf_ found in our study, both cultivars should be classified as relatively anisohydric as the slope of the relationship between Ψ_PD_ and *E* was not different (Figure 2B). However, *g*_s_ was differentially regulated, as shown by the association between *g*_s_ and Ψ_PD_, where Grenache had a steeper slope than Syrah (Figure 2A). Given that the root and plant hydraulic conductances in Grenache were closely correlated to *g*_s_, it is possible to consider an isohydrodynamic behavior in Grenache in which a strong stomatal control maintains relatively constant internal water potential gradients but, at the same time allows Ψ_leaf_ to fluctuate in synchrony with Ψ_PD_. This new perspective of water transport regulation in plants was examined in a study where a theoretical framework based on the relationship between midday and predawn leaf water potentials was used to characterize plant responses to drought (Martínez-Vilalta et al., 2014). The continuum between isohydry and anisohydry requires further exploration, and their functional significance and mechanism remains under debate.

### Regulation of Hydraulic Conductance by AQPs Under Mild WD

**FIGURE 6:**
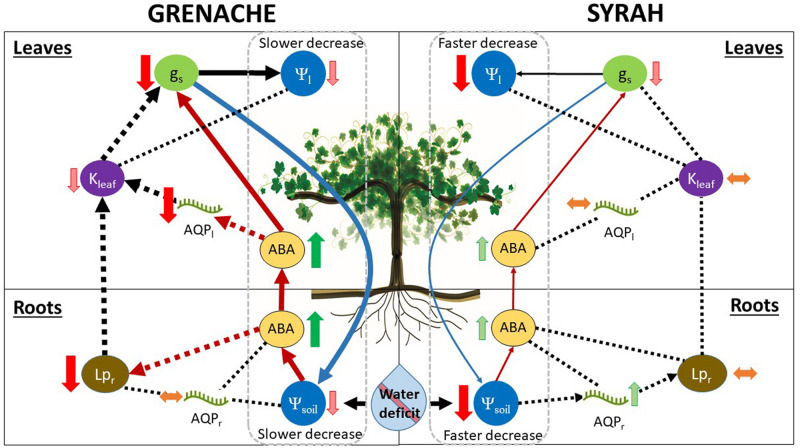
Summary of water deficit effects on water relations of the two grapevine cultivars, Grenache (left side) and Syrah (right side), in this study. Our observations support a model in which Grenache shows a stronger response to water deficit than Syrah through chemical (thick red arrows) and hydraulic pathways (thick black arrows) to limit water loss. In contrast, the cultivar Syrah has a weaker response (i.e., is less sensitive to water deficit than Grenache), which was mainly mediated through less responsive chemical pathway (thin red arrows), leading to greater water loss. Our observations do not support a strict iso-anisohydric distinction between the two cultivars, but rather a hydrodynamic behavior in which strong fluctuations in leaf water potential (Ψ_l__eaf_) are avoided by a tight control of hydraulic pathways. In Grenache, relatively strong control of water loss under water deficit is achieved in the following manner: Even a small decline in soil water potential (Ψ_soil_) leads to rapid synthesis of abscisic acid (ABA), causing strong reduction of root hydraulic conductivity (*Lp*_r_; thick dashed red arrow), as well as downregulation of some AQPs (transcripts) in leaves (AQP_l_) and, ultimately, decreased stomatal conductance (g_s_) through chemical regulation by ABA (thick red arrows). Our data did not support the hypothesis that (a reduction of) *Lp*_r_ was transcriptionally regulated by the select root aquaporins (AQP_r_) analyzed in this study. Alternative pathways of *Lp*_r_ regulation are possible that do not directly involve either ABA or AQP_r_, e.g., root suberisation, xylem embolisms. Additionally to chemical regulation, hydraulic regulation (thick black dashed arrows) may occur as a feed-forward signal, where reductions of *Lp*_r_ and leaf hydraulic conductance (K_leaf_) may induce stomatal closure. Stronger control over stomatal conductance in Grenache would lead to reduced water loss and, therefore, slower decrease of both Ψ_leaf_ and Ψ_soil_ (thick blue arrow). In Syrah, the control mechanisms for water loss under soil moisture deficit are less responsive than Grenache: Even a larger decline in Ψ_soil_ leads to only a small increase in the synthesis of ABA. A reduction in g_s_ appears to be mediated via the chemical pathway (thin red arrows), albeit less significantly compared to Grenache. The increase in ABA had no observable effect on *Lp*_r_, K_leaf_, or select AQP_l_ transcripts. Therefore, control of g_s_ via the hydraulic pathway (thin black dashed arrows) seems less likely. Weaker control of g_s_ in Syrah (compared to Grenache) resulted in greater water loss leading to greater reductions of both Ψ_leaf_ and Ψ_soil_ (thin blue arrow).

Aquaporins play a major role in the regulation of hydraulic conductance at the cellular level. As suggested by Vandeleur et al. (2014), AQPs are likely involved in the regulation of *Lp*_r_ in response to changes in *E*, which was explained by shoot-to-root signaling. Pou et al. (2013) found in grapevine that the expression of *TIP2*;*1* in the leaf was well-correlated to changes in *g*_s_ during drought and rehydration. In Touriga Nacional grapevines, Zarrouk et al. (2015) found that specific AQPs were downregulated in the roots of water stressed vines concurrent with decreases in *Lp_*r*_*, whereas the opposite trend was observed in the leaves: an upregulation of specific AQPs without changes in *K*_leaf_. In the present study, three of the AQPs examined (*PIP1;1*, *PIP2*;*1*, *TIP2*;*1*) were correlated with *K*_leaf_ in both cultivars: they were down-regulated under WD conditions in Grenache and no changes were observed for Syrah following a similar response of *K*_leaf_ in each cultivar (Figures 5C,E,G). This difference between Grenache and Syrah could explain different responses in hydraulic conductance to changes in *E*. Therefore, stronger leaf hydraulic control in Grenache could be explained by stronger regulation of AQP transcripts. Previous studies have observed an overexpression of *SlTIP2*;*2* in tomato that resulted in higher *E* in the transgenic plants (Sade et al., 2009). According to the research of Shatil-Cohen et al. (2011) and Pantin et al. (2013), AQPs modulate *K*_leaf_, e.g., via the bundle sheath-mesophyll-continuum, which would send a feed-forward signal to stomata. Hence, two different hypotheses exist for the relationship between *E* and hydraulic conductance. Either (i) changes in *E* affect hydraulic conductance through the function of AQPs, or, (ii) a hydraulic feed-forward signal mediated by AQPs affects *g*_s_. In the first case, a differential stomatal response could require alternative hydraulic regulation via AQPs, which would be the case in Grenache in the present study. In the second case, alternative hydraulic regulation by AQPs, potentially via ABA, could affect the differential stomatal behavior through feed-forward signaling to stomata. At the root level, a different situation was observed for both cultivars as the decrease in *Lp*_r_ under WD for Grenache was not followed by changes in root AQP expression levels indicating that such *Lp*_r_ reduction was mainly produced via the apoplastic pathway. In contrast, in Syrah, an up-regulation of the root AQPs *PIP1;1* and *PIP2*;*1* was observed despite constant *Lp*_r_ suggesting a contribution of AQPs to water transport via the symplastic pathway under WD conditions. This observation is in agreement with the findings of Vandeleur et al. (2014) who observed a contribution of AQPs at the root level in Chardonnay, but not in Grenache, after a WD was imposed. A summary of our findings is provided in the schematic below (Figure 6). As the expression of AQPs has been shown to be highly variable depending on environmental and experimental conditions, the involvement of these water channel proteins in the regulation of stomata under drought remains unclear requiring further investigation.

## Conclusion

A mild WD (Ψ_PD_ ∼−0.6 MPa) imposed on two grapevine cultivars showed a stronger reduction of *g*_s_ in Grenache as compared to Syrah. This response was supported by the higher sensitivity of Grenache to ABA concentration in the xylem sap. Despite this, these observations do not necessarily indicate a more isohydric behavior in Grenache than in Syrah since (i) the correlation between Ψ_PD_ and Ψ_leaf_ was similar for both cultivars; and, (ii) the responses observed under the range of Ψ_PD_ examined here might not apply under a more severe WD. In Grenache, the plant (*K*_plant_) and particularly the root (*Lp*_r_) hydraulic conductances showed a stronger response to the transpiration rate (*E*) than Syrah indicating potentially higher leaf-to-root communication in this cultivar.

## Data Availability Statement

All datasets generated for this study are included in the article/Supplementary Material.

## Author Contributions

SD, VP, and ST conceived the experiment. SD, JS, VP, ST, SR, WS, and FD conducted the experiments. SD and JS wrote the manuscript. SD, JS, VP, and ST edited the manuscript.

## Conflict of Interest

The authors declare that the research was conducted in the absence of any commercial or financial relationships that could be construed as a potential conflict of interest.

## References

[B1] Andersen C. L. Jensen J. L. Orntoft T. F. (2004). Normalization of real-time quantitative reverse transcription-PCR data: a model-based variance estimation approach to identify genes suited for normalization, applied to bladder and colon cancer data sets. *Cancer Res.* 64 5245–5250. 10.1158/0008-5472.can-04-0496 15289330

[B2] Aphalo P. J. Jarvis P. G. (1991). Do stomata respond to relative humidity? *Plant Cell Environ.* 14 127–132. 10.1111/j.1365-3040.1991.tb01379.x

[B3] Aroca R. Ferrante A. Vernieri P. Chrispeels M. J. (2006). Drought, abscisic acid and transpiration rate effects on the regulation of PIP aquaporin gene expression and abundance in *Phaseolus vulgaris* plants. *Ann. Bot.* 98 1301–1310. 10.1093/aob/mcl219 17028296 PMC2803586

[B4] Charrier G. Delzon S. Domec J. C. Zhang L. Delmas C. E. L. Merlin I. (2018). Drought will not leave your glass empty: low risk of hydraulic failure revealed by long-term drought observations in world’s top wine regions. *Sci. Adv.* 4:eaao6969. 10.1126/sciadv.aao6969 29404405 PMC5796794

[B5] Chaumont F. Tyerman S. D. (2014). Aquaporins: highly regulated channels controlling plant water relations. *Plant Physiol.* 164 1600–1618. 10.1104/pp.113.233791 24449709 PMC3982727

[B6] Chaves M. M. Zarrouk O. Francisco R. Costa J. M. Santos T. Regalado A. P. (2010). Grapevine under deficit irrigation: hints from physiological and molecular data. *Ann. Bot. Lon.* 105 661–676. 10.1093/Aob/Mcq030 20299345 PMC2859908

[B7] Christmann A. Weiler E. W. Steudle E. Grill E. (2007). A hydraulic signal in root-to-shoot signalling of water shortage. *Plant J.* 52 167–174. 10.1111/j.1365-313X.2007.03234.x 17711416

[B8] Comstock J. P. (2002). Hydraulic and chemical signalling in the control of stomatal conductance and transpiration. *J. Exp. Bot.* 53 195–200. 10.1093/jexbot/53.367.195 11807122

[B9] Coupel-Ledru A. Lebon É Christophe A. Doligez A. Cabrera-Bosquet L. Péchier P. (2014). Genetic variation in a grapevine progeny (*Vitis vinifera* L. cvs Grenache × Syrah) reveals inconsistencies between maintenance of daytime leaf water potential and response of transpiration rate under drought. *J. Exp. Bot.* 65 6205–6218. 10.1093/jxb/eru228 25381432 PMC4223985

[B10] Coupel-Ledru A. Tyerman S. Masclef D. Lebon E. Christophe A. Edwards E. J. (2017). Abscisic acid down-regulates hydraulic conductance of grapevine leaves in isohydric genotypes only. *Plant Physiol.* 175 1121–1134. 10.1104/pp.17.00698 28899961 PMC5664463

[B11] Cutler A. J. Krochko J. E. (1999). Formation and breakdown of ABA. *Trends Plant Sci.* 4 472–478. 10.1016/S1360-1385(99)01497-1 10562731

[B12] DeGaris K. A. (2016). *Direct and Indirect Influences of Water Deficit on Salt Uptake, Ion Accumulation and Root-Shoot Interactions of Grapevines.* Ph.D., The University of Adelaide, Adelaide.

[B13] Dodd I. C. (2003). Hormonal interactions and stomatal responses. *J. Plant Growth Regul.* 22 32–46. 10.1007/s00344-003-0023-x

[B14] Dodd I. C. (2005). Root-to-shoot signalling: assessing the roles of ‘up’ in the up and down world of long-distance signalling in planta. *Plant Soil* 274 251–270. 10.1007/s11104-004-0966-0

[B15] Flexas J. Scoffoni C. Gago J. Sack L. (2013). Leaf mesophyll conductance and leaf hydraulic conductance: an introduction to their measurement and coordination. *J. Exp. Bot.* 64 3965–3981. 10.1093/Jxb/Ert319 24123453

[B16] Franks P. J. Drake P. L. Froend R. H. (2007). Anisohydric but isohydrodynamic: seasonally constant plant water potential gradient explained by a stomatal control mechanism incorporating variable plant hydraulic conductance. *Plant Cell Environ.* 30 19–30. 10.1111/j.1365-3040.2006.01600.x 17177873

[B17] Galmes J. Pou A. Alsina M. M. Tomas M. Medrano H. Flexas J. (2007). Aquaporin expression in response to different water stress intensities and recovery in Richter-110 (*Vitis* sp.): relationship with ecophysiological status. *Planta* 226 671–681. 10.1007/s00425-007-0515-1 17447082

[B18] Gerzon E. Biton I. Yaniv Y. Zemach H. Netzer Y. Schwartz A. (2015). Grapevine anatomy as a possible determinant of isohydric or anisohydric behavior. *Am. J. Enol. Viticult.* 66 340–347. 10.5344/ajev.2015.14090

[B19] Hochberg U. Bonel A. G. David-Schwartz R. Degu A. Fait A. Cochard H. (2017). Grapevine acclimation to water deficit: the adjustment of stomatal and hydraulic conductance differs from petiole embolism vulnerability. *Planta* 245 1091–1104. 10.1007/s00425-017-2662-3 28214919 PMC5432590

[B20] Holbrook N. M. Shashidhar V. R. James R. A. Munns R. (2002). Stomatal control in tomato with ABA-deficient roots: response of grafted plants to soil drying. *J. Exp. Bot.* 53 1503–1514. 10.1093/jexbot/53.373.1503 12021298

[B21] Kudoyarova G. Veselova S. Hartung W. Farhutdinov R. Veselov D. Sharipova G. (2011). Involvement of root ABA and hydraulic conductivity in the control of water relations in wheat plants exposed to increased evaporative demand. *Planta* 233 87–94. 10.1007/s00425-010-1286-7 20924765

[B22] Laur J. Hacke U. G. (2013). Transpirational demand affects aquaporin expression in poplar roots. *J. Exp. Bot.* 64 2283–2293. 10.1093/jxb/ert096 23599275 PMC3654427

[B23] Lenth R. (2019). *Emmeans: Estimated Marginal Means, Aka Least-Squares Means. R Package Version* 1.3.3. Available online at: https://CRAN.R-project.org/package=emmeans.

[B24] Li G. Boudsocq M. Hem S. Vialaret J. Rossignol M. Maurel C. (2015). The calcium-dependent protein kinase CPK7 acts on root hydraulic conductivity. *Plant Cell Environ.* 38 1312–1320. 10.1111/pce.12478 25366820

[B25] Lovisolo C. Perrone I. Carra A. Ferrandino A. Flexas J. Medrano H. (2010). Drought-induced changes in development and function of grapevine (*Vitis* spp.) organs and in their hydraulic and non-hydraulic interactions at the whole-plant level: a physiological and molecular update. *Funct. Plant Biol.* 37 98–116. 10.1071/FP09191

[B26] Martínez-Vilalta J. Poyatos R. Aguadé D. Retana J. Mencuccini M. (2014). A new look at water transport regulation in plants. *New Phytol.* 204 105–115. 10.1111/nph.12912 24985503

[B27] Maurel C. Simonneau T. Sutka M. (2010). The significance of roots as hydraulic rheostats. *J. Exp. Bot.* 61 3191–3198. 10.1093/jxb/erq150 20522526

[B28] McAdam S. A. Manzi M. Ross J. J. Brodribb T. J. Gómez-Cadenas A. (2016). Uprooting an abscisic acid paradigm: shoots are the primary source. *Plant Signal. Behav.* 11 652–659. 10.1080/15592324.2016.1169359 PMC4973758 27031537

[B29] Medrano H. Escalona J. M. Bota J. Gulías J. Flexas J. (2002). Regulation of photosynthesis of C3 plants in response to progressive drought: stomatal conductance as a reference parameter. *Ann. Bot.* 89 895–905. 10.1093/aob/mcf079 12102515 PMC4233802

[B30] Mekonnen D. W. Flugge U. I. Ludewig F. (2016). Gamma-aminobutyric acid depletion affects stomata closure and drought tolerance of *Arabidopsis thaliana*. *Plant Sci.* 245 25–34. 10.1016/j.plantsci.2016.01.005 26940489

[B31] Nardini A. Tyree M. T. Salleo S. (2001). Xylem cavitation in the leaf of *Prunus laurocerasus* and its impact on leaf hydraulics. *Plant Physiol.* 125 1700–1709. 10.1104/pp.125.4.1700 11299351 PMC88827

[B32] Pantin F. Monnet F. Jannaud D. Costa J. M. Renaud J. Muller B. (2013). The dual effect of abscisic acid on stomata. *New Phytol.* 197 65–72. 10.1111/nph.12013 23106390

[B33] Parent B. Hachez C. Redondo E. Simonneau T. Chaumont F. Tardieu F. (2009). Drought and abscisic acid effects on aquaporin content translate into changes in hydraulic conductivity and leaf growth rate: a trans-scale approach. *Plant Physiol.* 149 2000–2012. 10.1104/pp.108.130682 19211703 PMC2663730

[B34] Pfaffl M. W. (2001). A new mathematical model for relative quantification in real-time RT-PCR. *Nucleic Acids Res.* 29:e45. 10.1093/nar/29.9.e45 11328886 PMC55695

[B35] Pou A. Medrano H. Flexas J. Tyerman S. D. (2013). A putative role for TIP and PIP aquaporins in dynamics of leaf hydraulic and stomatal conductances in grapevine under water stress and re-watering. *Plant Cell Environ.* 36 828–843. 10.1111/pce.12019 23046275

[B36] Prado K. Boursiac Y. Tournaire-Roux C. Monneuse J. M. Postaire O. Da Ines O. (2013). Regulation of *Arabidopsis* leaf hydraulics involves light-dependent phosphorylation of aquaporins in veins. *Plant Cell* 25 1029–1039. 10.1105/tpc.112.108456 23532070 PMC3634675

[B37] Prado K. Maurel C. (2013). Regulation of leaf hydraulics: from molecular to whole plant levels. *Front. Plant Sci.* 4:255. 10.3389/fpls.2013.00255 23874349 PMC3711007

[B38] Prieto J. A. Lebon É Ojeda H. (2010). Stomatal behavior of different grapevine cultivars in response to soil water status and air water vapor pressure deficit. *OENO One* 44 9–20. 10.20870/oeno-one.2010.44.1.1459

[B39] Rossdeutsch L. Edwards E. Cookson S. J. Barrieu F. Gambetta G. A. Delrot S. (2016). ABA-mediated responses to water deficit separate grapevine genotypes by their genetic background. *BMC Plant Biol.* 16:91. 10.1186/s12870-016-0778-4 27091220 PMC4836075

[B40] Sack L. Holbrook N. M. (2006). Leaf hydraulics. *Annu. Rev. Plant Biol.* 57 361–381. 10.1146/annurev.arplant.56.032604.144141 16669766

[B41] Sack L. Melcher P. J. Zwieniecki M. A. Holbrook N. M. (2002). The hydraulic conductance of the angiosperm leaf lamina: a comparison of three measurement methods. *J. Exp. Bot.* 53 2177–2184. 10.1093/jxb/erf069 12379784

[B42] Sade N. Vinocur B. J. Diber A. Shatil A. Ronen G. Nissan H. (2009). Improving plant stress tolerance and yield production: is the tonoplast aquaporin SlTIP2;2 a key to isohydric to anisohydric conversion? *New Phytol.* 181 651–661. 10.1111/j.1469-8137.2008.02689.x 19054338

[B43] Sakurai-Ishikawa J. Murai-Hatano M. Hayashi H. Ahamed A. Fukushi K. Matsumoto T. (2011). Transpiration from shoots triggers diurnal changes in root aquaporin expression. *Plant Cell Environ.* 34 1150–1163. 10.1111/j.1365-3040.2011.02313.x 21414014

[B44] Salleo S. Nardini A. Pitt F. Gullo M. A. L. (2000). Xylem cavitation and hydraulic control of stomatal conductance in Laurel (*Laurus nobilis* L.). *Plant Cell Environ.* 23 71–79. 10.1046/j.1365-3040.2000.00516.x

[B45] Scharwies J. D. Tyerman S. D. (2016). Comparison of isohydric and anisohydric *Vitis vinifera* L. cultivars reveals a fine balance between hydraulic resistances, driving forces and transpiration in ripening berries. *Funct. Plant Biol.* 44 322–336. 10.1071/FP16010 32480567

[B46] Schroeder J. I. Allen G. J. Hugouvieux V. Kwak J. M. Waner D. (2001). Guard cell signal transduction. *Annu. Rev. Plant Physiol. Plant Mol. Biol.* 52 627–658. 11337411 10.1146/annurev.arplant.52.1.627

[B47] Schultz H. R. (2003). Differences in hydraulic architecture account for near-isohydric and anisohydric behaviour of two field-grown *Vitis vinifera* L. cultivars during drought. *Plant Cell Environ.* 26 1393–1405. 10.1046/j.1365-3040.2003.01064.x

[B48] Selim M. Legay S. Berkelmann-Lohnertz B. Langen G. Kogel K. H. Evers D. (2012). Identification of suitable reference genes for real-time RT-PCR normalization in the grapevine-downy mildew pathosystem. *Plant Cell Rep.* 31 205–216. 10.1007/s00299-011-1156-1 22006104

[B49] Shatil-Cohen A. Attia Z. Moshelion M. (2011). Bundle-sheath cell regulation of xylem-mesophyll water transport via aquaporins under drought stress: a target of xylem-borne ABA? *Plant J.* 67 72–80. 10.1111/j.1365-313X.2011.04576.x 21401747

[B50] Shelden M. C. Vandeleur R. Kaiser B. N. Tyerman S. D. (2017). A comparison of petiole hydraulics and aquaporin expression in an anisohydric and isohydric cultivar of grapevine in response to water-stress induced cavitation. *Front. Plant Sci.* 8:1893. 10.3389/fpls.2017.01893 29163613 PMC5681967

[B51] Simonin K. A. Burns E. Choat B. Barbour M. M. Dawson T. E. Franks P. J. (2015). Increasing leaf hydraulic conductance with transpiration rate minimizes the water potential drawdown from stem to leaf. *J. Exp. Bot.* 66 1303–1315. 10.1093/jxb/eru481 25547915 PMC4339593

[B52] Soar C. J. Speirs J. Maffei S. M. Penrose A. B. McCarthy M. G. Loveys B. R. (2006). Grape vine varieties Shiraz and Grenache differ in their stomatal response to VPD: apparent links with ABA physiology and gene expression in leaf tissue. *Austr. J. Grape Wine Res.* 12 2–12. 10.1111/j.1755-0238.2006.tb00038.x

[B53] Speirs J. Binney A. Collins M. Edwards E. Loveys B. (2013). Expression of ABA synthesis and metabolism genes under different irrigation strategies and atmospheric VPDs is associated with stomatal conductance in grapevine (*Vitis vinifera* L. cv Cabernet Sauvignon). *J. Exp. Bot.* 64 1907–1916. 10.1093/jxb/ert052 23630325 PMC3638820

[B54] Steudle E. (2000). Water uptake by plant roots: an integration of views. *Plant Soil* 226 45–56. 10.1023/a:1026439226716

[B55] Takahashi F. Suzuki T. Osakabe Y. Betsuyaku S. Kondo Y. Dohmae N. (2018). A small peptide modulates stomatal control via abscisic acid in long-distance signalling. *Nature* 556:235. 10.1038/s41586-018-0009-2 29618812

[B56] Tardieu F. Lafarge T. Simonneau T. (1996). Stomatal control by fed or endogenous xylem ABA in sunflower: interpretation of correlations between leaf water potential and stomatal conductance in anisohydric species. *Plant Cell Environ.* 19 75–84. 10.1111/j.1365-3040.1996.tb00228.x

[B57] Tardieu F. Simonneau T. (1998). Variability among species of stomatal control under fluctuating soil water status and evaporative demand: modelling isohydric and anisohydric behaviours. *J. Exp. Bot.* 49 419–432. 10.1093/jexbot/49.suppl_1.419

[B58] Thompson A. J. Andrews J. Mulholland B. J. McKee J. M. T. Hilton H. W. Horridge J. S. (2007). Overproduction of abscisic acid in tomato increases transpiration efficiency and root hydraulic conductivity and influences leaf expansion. *Plant Physiol.* 143 1905–1917. 10.1104/pp.106.093559 17277097 PMC1851808

[B59] Tombesi S. Nardini A. Frioni T. Soccolini M. Zadra C. Farinelli D. (2015). Stomatal closure is induced by hydraulic signals and maintained by ABA in drought-stressed grapevine. *Sci. Rep.* 5:12449. 10.1038/srep12449 26207993 PMC4513549

[B60] Tornroth-Horsefield S. Wang Y. Hedfalk K. Johanson U. Karlsson M. Tajkhorshid E. (2006). Structural mechanism of plant aquaporin gating. *Nature* 439 688–694. 10.1038/nature04316 16340961

[B61] Tournaire-Roux C. Sutka M. Javot H. Gout E. Gerbeau P. Luu D.-T. (2003). Cytosolic pH regulates root water transport during anoxic stress through gating of aquaporins. *Nature* 425 393–397. 10.1038/nature01853 14508488

[B62] Tyree M. T. Sperry J. S. (1989). Vulnerability of xylem to cavitation and embolism. *Ann. Rev. Plant Physiol. Plant Mol. Biol.* 40 19–38. 10.1146/annurev.pp.40.060189.000315

[B63] Vandeleur R. K. Mayo G. Shelden M. C. Gilliham M. Kaiser B. N. Tyerman S. D. (2009). The role of plasma membrane intrinsic protein aquaporins in water transport through roots: diurnal and drought stress responses reveal different strategies between isohydric and anisohydric cultivars of grapevine. *Plant Physiol.* 149 445–460. 10.1104/pp.108.128645 18987216 PMC2613730

[B64] Vandeleur R. K. Sullivan W. Athman A. Jordans C. Gilliham M. Kaiser B. N. (2014). Rapid shoot-to-root signalling regulates root hydraulic conductance via aquaporins. *Plant Cell Environ.* 37 520–538. 10.1111/pce.12175 23926961

[B65] Wilkinson S. Bacon M. A. Davies W. J. (2007). Nitrate signalling to stomata and growing leaves: interactions with soil drying, ABA, and xylem sap pH in maize. *J. Exp. Bot.* 58 1705–1716. 10.1093/jxb/erm021 17374875

[B66] Zarrouk O. Garcia-Tejero I. Pinto C. Genebra T. Sabir F. Prista C. (2015). Aquaporins isoforms in cv. Touriga Nacional grapevine under water stress and recovery—Regulation of expression in leaves and roots. *Agric. Water Manage.* 164 167–175. 10.1016/j.agwat.2015.08.013

