# Supplementary figures and images for: Comparing Hydraulics Between Two Grapevine Cultivars Reveals Differences in Stomatal Regulation Under Water Stress and Exogenous ABA Applications
Source: Front Plant Sci. 2020 Jun 19;11:705. doi: 10.3389/fpls.2020.00705 (PMC7316991; doi:10.3389/fpls.2020.00705)

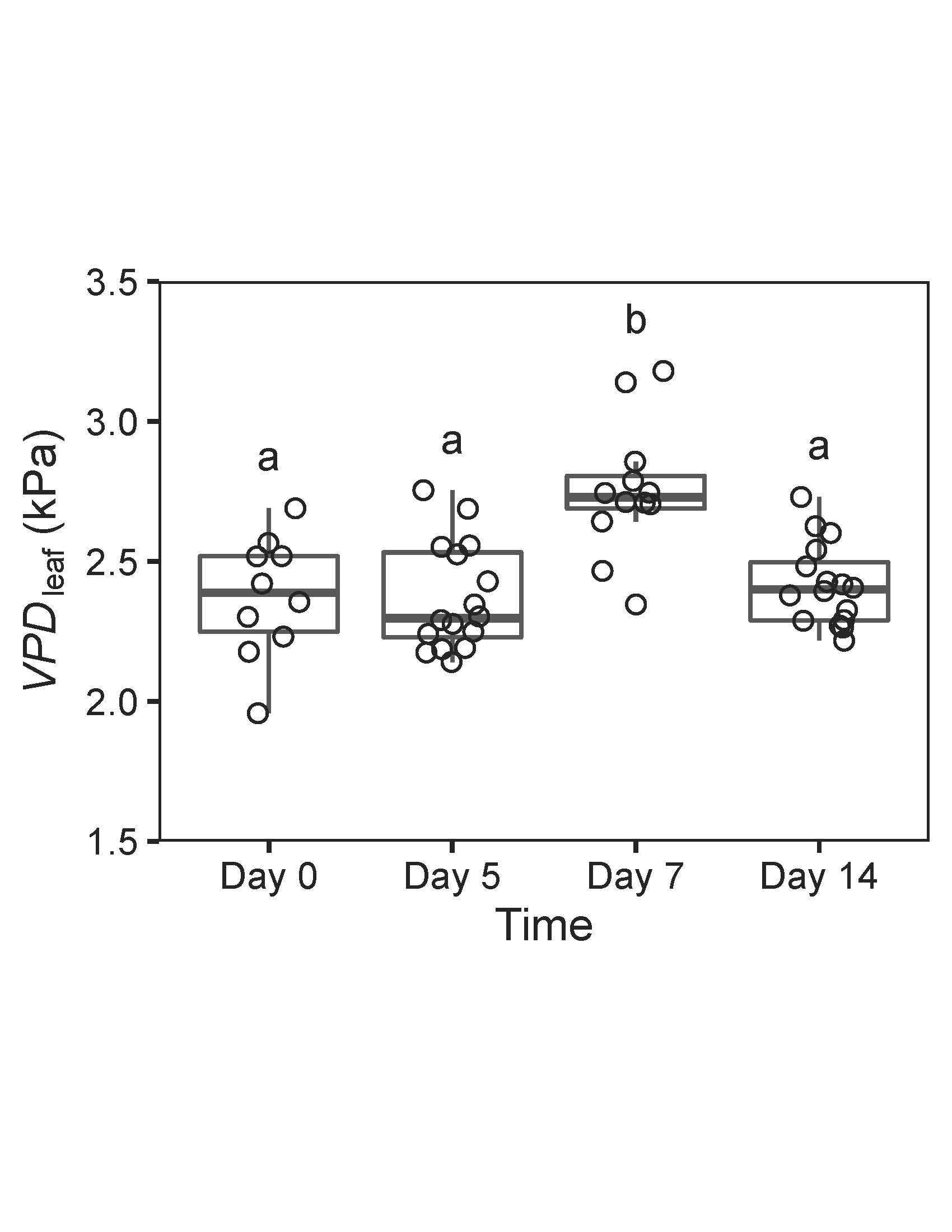

Supplement: Supplementary file 1 [file Image_1.JPEG]

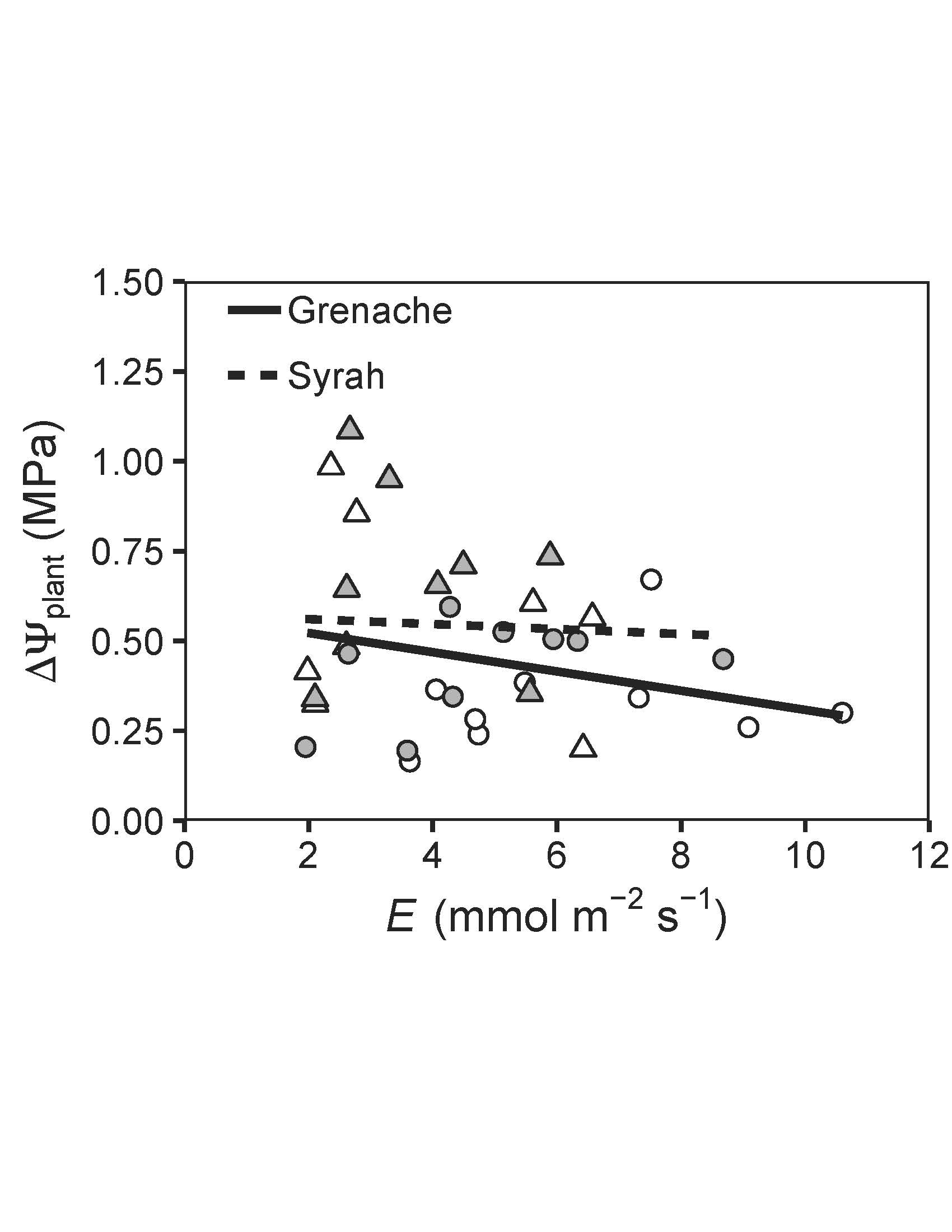

Supplement: Supplementary file 2 [file Image_2.JPEG]

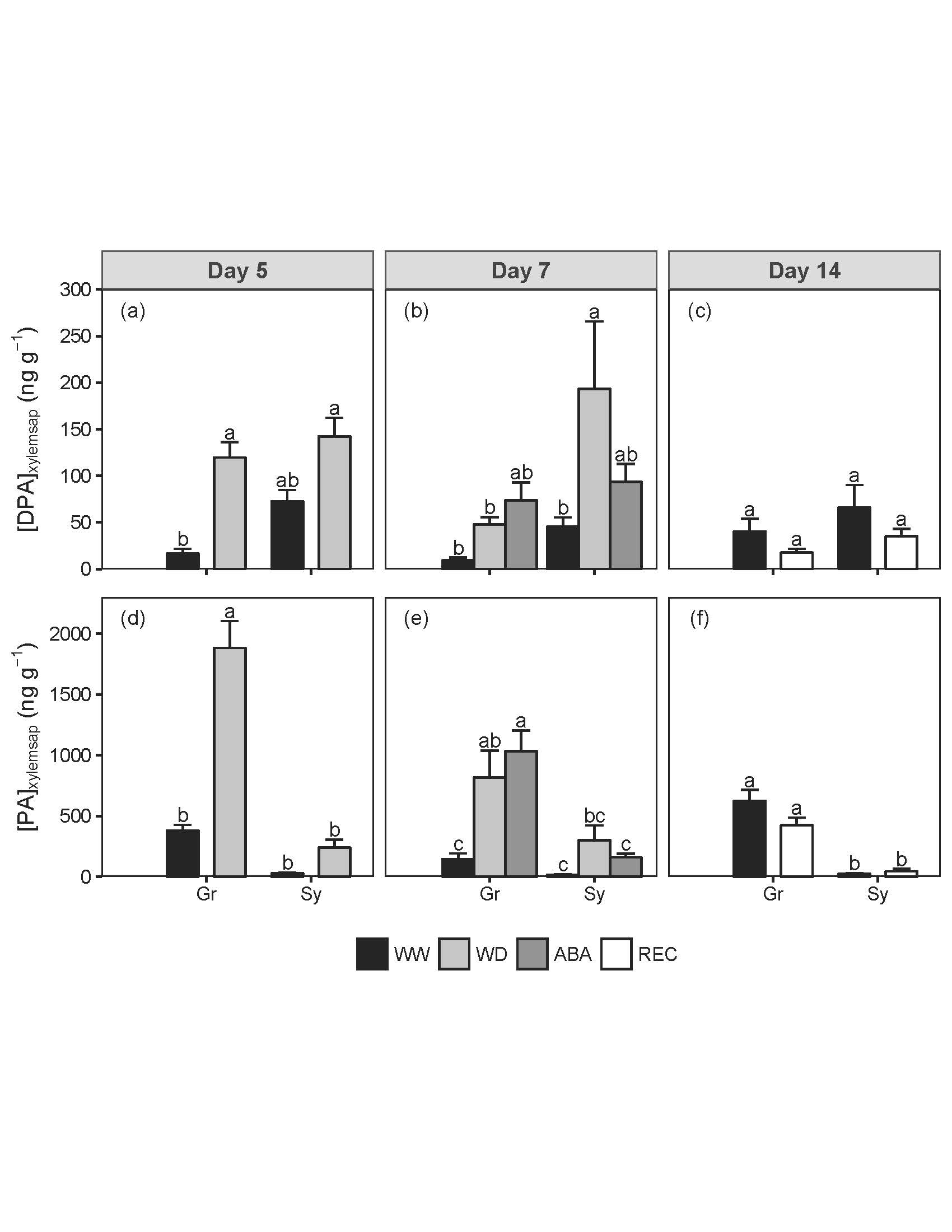

Supplement: Supplementary file 3 [file Image_3.JPEG]

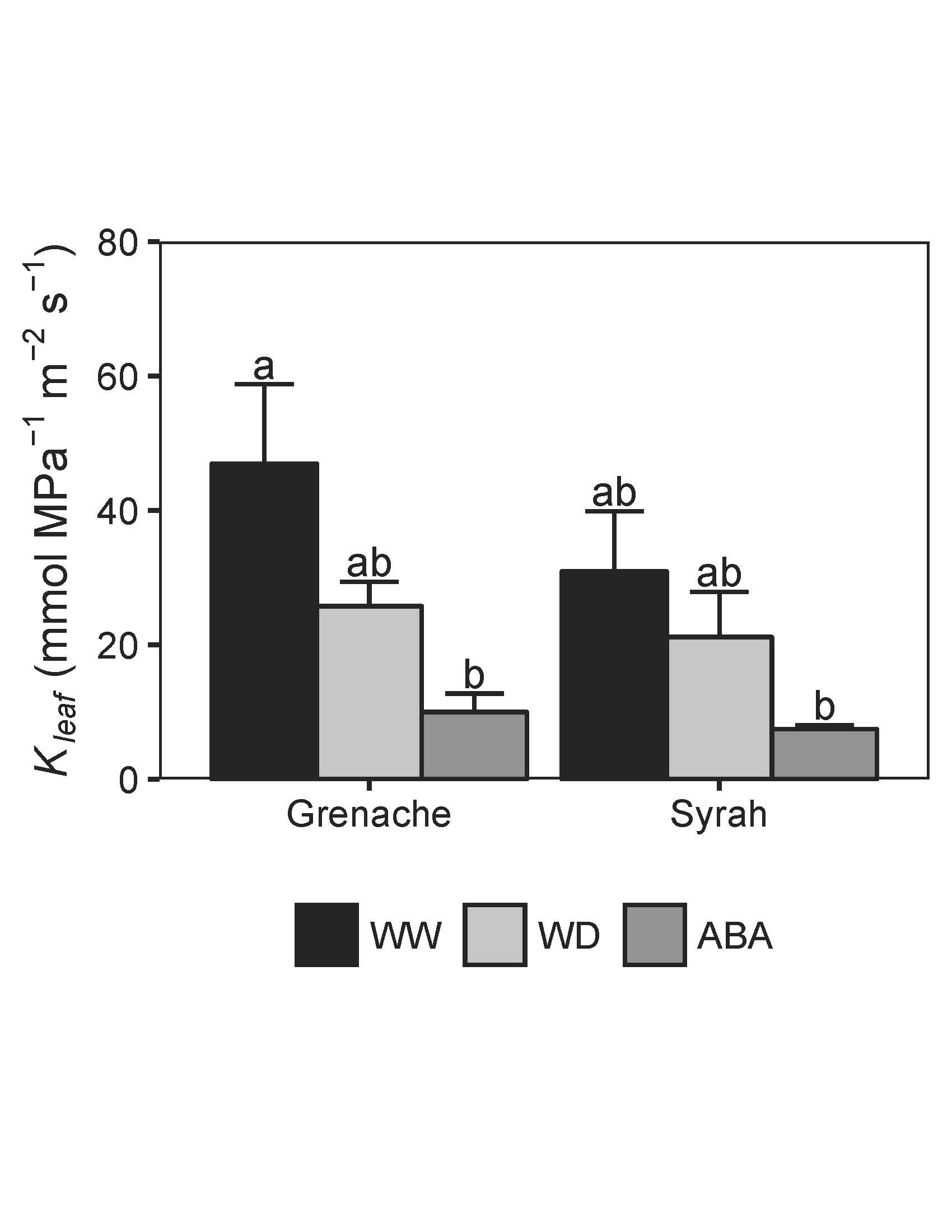

Supplement: Supplementary file 4 [file Image_4.JPEG]
