# Supplementary material for: Comparing Hydraulics Between Two Grapevine Cultivars Reveals Differences in Stomatal Regulation Under Water Stress and Exogenous ABA Applications
Source: Front Plant Sci. 2020 Jun 19;11:705. doi: 10.3389/fpls.2020.00705 (PMC7316991; doi:10.3389/fpls.2020.00705)
Supplement: Supplementary file 5 [file Table_1.docx]

**Table S1.** Sequences of gene-specific primers to amplify reference and aquaporin genes in RT-qPCR.

| ***Vitis* Gene** | **Sequence** |
| --- | --- |
| *ELF* | Forward; 5’-CGGGCAAGAGATACCTCAAT-3’ |
|  | Reverse; 5’-AGAGCCTCTCCCTCAAAAGG-3’ |
| *PIP1;1* | Forward; 5’-TGGTGCGGGTGTAGTGAAGG-3’ |
|  | Reverse; 5’-AGACAGTGTAGACAAGGACGAAGG-3’ |
| *PIP2;1* | Forward; 5’-GGCATTTCTGGGGGACACAT-3’ |
|  | Reverse; 5’-CTTTGACGAGACCCACACCA-3’ |
| *TIP2;1* | Forward; 5’-TTAACCCTGCGGTGACCTTC-3’ |
|  | Reverse; 5’-TCAATGACTCCAACCCCAGC-3’ |
