# Supplementary material for: Comparing Hydraulics Between Two Grapevine Cultivars Reveals Differences in Stomatal Regulation Under Water Stress and Exogenous ABA Applications
Source: Front Plant Sci. 2020 Jun 19;11:705. doi: 10.3389/fpls.2020.00705 (PMC7316991; doi:10.3389/fpls.2020.00705)
Supplement: Supplementary file 6 [file Presentation_1.docx]

**Figure S1.** *VPD*_leaf_ measured by the IRGA during the different days of the experiment. Different letters indicate statistically significant differences (*P* ≤ 0.05).

**Figure S2.** Relationship between transpiration (*E*) and the plant water potential gradient (ΔΨ_plant_ = Ψ_PD_ – Ψ_leaf_) measured for well-watered (circles) and water deficit vines (triangles) from the cultivars Grenache (open symbols) and Syrah (filled symbols). Linear regressions fitted to the data were not significant and no differences were found between the two cultivars.

**Figure S3.** Abscisic acid catabolites, dihydrophaseic acid (DPA ; a-c) and phaseic acid (PA; d-f), measured in the xylem sap of Grenache and Syrah grapevines under well-watered (WW), mild water deficit (WD), exogenous application of abscisic acid (ABA), and recovery from water stress (REC) at different sampling days along the experiment. Values are means ± SE (n = 3-5). Different letters indicate statistically significant differences (*P* ≤ 0.05) across all treatments and cultivars within the day.

**Figure S4.** Comparison of *K_leaf_* between grapevines of the cultivars Grenache and Syrah under well-watered (WW), mild water deficit (WD), and exogenous application of abscisic acid (ABA). Data from Day 5 and Day 7 were pooled. Values are means ± SE (n = 4 - 8). Different letters indicate statistically significant differences (*P* ≤ 0.05) across all treatments and cultivars.
